# Clinical aspects of cervical insufficiency

**DOI:** 10.1186/1471-2393-7-S1-S17

**Published:** 2007-06-01

**Authors:** Frederik K Lotgering

**Affiliations:** 1 Department of Obstetrics and Gynecology, 791 Radboud University Nijmegen Medical Centre, PO Box 1901, 6500 HB Nijmegen, the Netherlands

## Abstract

Fetal loss is a painful experience. A history of second or early third trimester fetal loss, after painless dilatation of the cervix, prolapse or rupture of the membranes, and expulsion of a live fetus despite minimal uterine activity, is characteristic for cervical insufficiency. In such cases the risk of recurrence is high, and a policy of prophylactic cerclage may be safer than one of serial cervical length measurements followed by cerclage, tocolysis and bed rest in case of cervical shortening or dilatation. In low risk cases, however, prophylactic cerclage is not useful. There is a need for more basic knowledge of cervical ripening, objective assessment of cervical visco-elastic properties, and randomized controlled trials of technical aspects of cervical cerclage (e.g. suturing technique).

## Background

Classic cervical insufficiency is a diagnosis, based on an obstetric history of *recurrent *second- or early third-trimester fetal loss, following painless cervical dilatation, prolapse or rupture of the membranes, and expulsion of a live fetus despite minimal uterine activity [1]. In the absence of the classic recurrence, the term cervical insufficiency is generally used as a work-diagnosis based on a *single *event with the same characteristic clinical history after exclusion of other possible causes of preterm delivery. In the absence of a second- or early third-trimester fetal loss, it is incorrect to use the term cervical insufficiency in connection with a short or traumatized cervix alone. High risk of cervical insufficiency may result from developmental abnormality (abnormal collagen or diethylstilbestrol exposure), previous surgery (amputation or exconization), and laceration by previous transvaginal cerclage or cervical rupture.

Observational studies show that in classical cases with a severely traumatized or virtually absent cervix, neonatal survival may be up to 93% after effective cerclage as compared to 27% before the cerclage [2]. Others regard the diagnosis of cervical insufficiency as elusive because of the lack of uniform diagnostic criteria and/or an objective diagnostic test [3], and cerclage therapy as ineffective because pooled data of randomized controlled trials show no reduction in fetal loss [4]. That raises the question if absence of proof from randomized controlled trials should be taken as proof of absence of reduction of fetal loss by cervical cerclage in cases at high risk for cervical insufficiency.

One may wonder why no large-scale randomized controlled trials have been performed to definitively prove the effectiveness of cervical cerclage, while there is such an obvious need for those studies. One reason could be that patients at high risk of yet another fetal loss are unwilling to give their consent to randomization after being informed that observational studies have shown approximately 90% infant viability after cerclage and a low rate of procedure related complications [3]. That may explain why studies of the effectiveness of cerclage have been relatively small scale and/or have not been performed on truly high-risk patients. Small-scale studies in relatively low-risk patients are little informative of the true value of cerclage, as the power is low and both arms of the studies will have relatively good outcome. Unfortunately, those studies carry the risk that the lack of effectiveness in a low-risk population is falsely extrapolated to high-risk patients who, in contrast to low-risk patients, were not studied and might well benefit from such a procedure.

The aim of this report is to determine if, from a clinical-mechanistic point of view, there is reason to question either the concept of cervical insufficiency or the efficacy of cerclage treatment in selected high-risk cases.

## Pathophysiology of premature cervical ripening

The physiological changes of cervical tissue remodeling, called ripening, as recently reviewed, are complex and incompletely understood [5]. What is know is that the uterine cervix is a dynamic anatomical structure that serves during most of gestation as a barrier between the fetus and its intra-uterine environment and the vagina as the portal to the outside world. During that time it is a firm structure that predominantly consists of collagen, but in the prelude to parturition the collagen is degraded and the cervix becomes soft and pliable enough to dilate. Imperfections in the process and/or timing of cervical ripening do occur, given the occurrence of preterm labor and dystocia in labor. Infection and inflammation are causally related to preterm labor and cervical ripening [6]. This relates to the cervical properties, as the chance of preterm delivery is inversely related to the length of the cervical canal [7], which contains mucus with antibacterial properties [8]. If the mechanical and/or antibacterial properties of the cervix are anatomically or functionally impaired, for example by intra-uterine exposure to diethylstilbestrol, or by surgery or trauma to the cervix, the remaining strength of the cervix may be insufficient to retain the pregnancy.

## Patient selection for cerclage

For obvious reasons, both women and doctors are unwilling to wait till the diagnosis of classic cervical insufficiency has been established by recurrence of fetal loss. In women considered to be at high risk for cervical incompetence, the recurrence risk of fetal loss without cerclage is not exactly known, due to lack of properly designed studies. Uncontrolled studies suggest that infant viability is about 25% without cerclage, whereas it is 75–90% after cerclage [3]. For that reason, in women with a history of classic cervical insufficiency prophylactic cerclage should be strongly considered. In case the work-diagnosis of cervical insufficiency is made after a single fetal loss, the effectiveness of prophylactic cervical cerclage may be questioned and the advantages and disadvantages should be carefully weighted against the other options. Without prophylactic cerclage, one accepts the risk that the cervix may open quite suddenly – within days after documented absence of funneling and a normal cervical length. The main alternative to prophylactic cerclage is a policy of serial cervical length measurements, followed after cervical shortening or dilatation by urgent or emergency cerclage with or without bulging of the membranes. Another alternative to prophylactic cerclage could be the cerclage pessary [9], but published experience is too limited to allow any conclusion on its effectiveness. In women at low risk for cervical insufficiency, prophylactic cerclage is of no proven benefit and should not be offered, regardless of cervical length by ultrasound [4].

## Technical aspects of cerclages

Once the conclusion has been reached that cervical cerclage is likely to benefit the patient at high or medium-high risk of cervical insufficiency, the question arises how and when to perform it.

**Figure 1:**
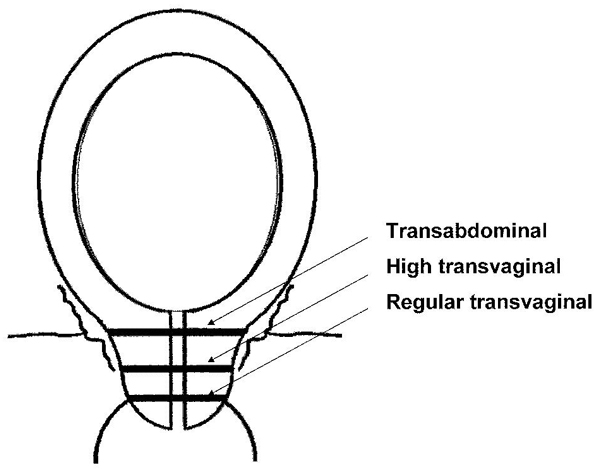
Three types of cerclage.

**Figure 2:**
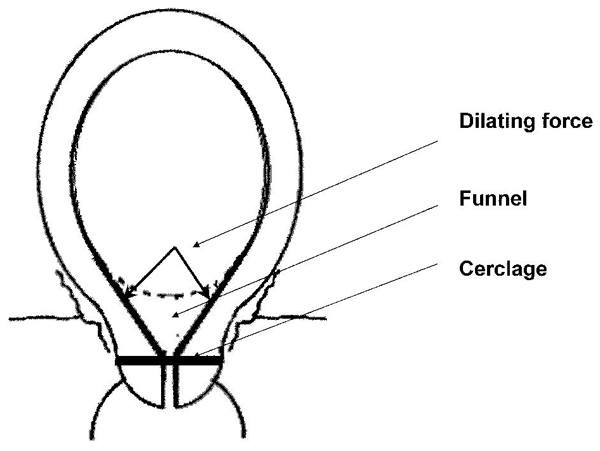
Funnelling.

Figure 1 shows the three main levels/types of cerclage: (1). regular transvaginal cerclage at the junction of cervix and fornix, (2) high-transvaginal cerclage after opening the fornix, and (3) transabdominal cerclage at the level of the internal cervical os. The effectiveness of these levels of cerclage has not been systematically studied. From a clinical/mechanical point of view, cervicoisthmic cerclage is superior to other cerclages as it is inserted at the level of the internal cervical os and therefore prevents funneling (opening of the cervical canal from the internal os). As illustrated in Figure 2, the presence of funneling is disadvantageous because any increase in intra-uterine pressure may, in the presence of funneling, exert a dilating force while the short remaining cervical length acts less as an antibacterial barrier and offers less mechanical strength. In contrast, from a surgical point of view transvaginal cerclages have the advantage over transabdominal cerclage, as the surgery is shorter and less challenging, the hospitalization is shorter, and there is no need for delivery by cesarean section as in transabdominal cerclage. Transabdominal cerclage should probably be performed only if adequate transvaginal cerclage is considered technically unfeasible or hazardous because of severe cervical defects [2].

**Figure 3:**
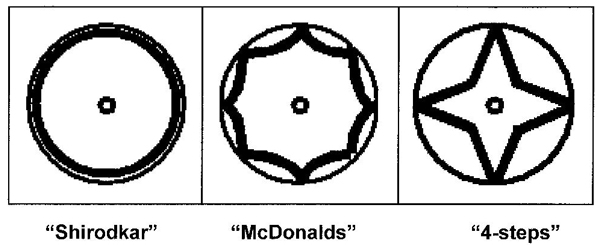
Three types of suturing.

Not only the position relative to the cervical canal, but also the position relative to the cervical tissue itself might affect the strength of the cerclage. Figure 3 shows three types of suturing: 1. around the cervix (modified Shirodkar), 2. with a series of small bites (modified McDonald), and 3. with 4 large bites of cervical tissue (4-steps). In one comparison, no significant difference in effectiveness was found between the Shirodkar and the McDonald technique [10]. From a mechanical point of view, the 4-steps method would seem to provide the better strength, because the band passes deeper through the tissue and the cervix is least likely to tear should uterine contractions appear.

Based on timing, one may differentiate between 1. prophylactic cerclage prior to conception, 2. prophylactic cerclage in pregnancy, 3. urgent cerclage after shortening of the cervix, and 4. emergency cerclage after exposure of the membranes. In high-risk patients, prophylactic cerclage has the advantage that one will not be surprised by yet another fetal loss or sudden shortening or opening of the cervix that requires urgent or emergency cerclage. An additional possible advantage of prophylactic cerclage could be that it may serve as an early warning system, as any serious force on it is likely to induce pain or blood loss, which would allow time to start tocolytic therapy.

Prophylactic transvaginal cerclage generally is an easy procedure, and morbidity is limited to hospital admission, mild pyrexia and tocolytic therapy [4]. Prophylactic transabdominal cerclage is more challenging, as one operates near the uterine vasculature. Some authors favor performance of transabdominal cerclage before conception, and the use of laparoscopic technique [11] has the advantages of minimally invasive surgery. However, an obvious disadvantage of preconception cerclage is that pregnancy may not occur, either deliberately or involuntarily, and published experience provides no evidence that preconception transabdominal cerclage is surgically easier or has fewer complications than transabdominal cerclage performed between 12 and 16 weeks gestation [2].

Emergency cerclages have traditionally been associated with a high risk of chorioamnionitis (up to 37%) and/or rupture of the membranes within 2 weeks of the operation (up to 65%), as a result of cervical shortening and exposure of the membranes to vaginal bacteria [3]. For that reason, a policy of serial cervical length measurements is an insecure alternative to prophylactic cerclage in high risk cases. However, recent small studies seem to suggest that emergency cerclage, in combination with antibiotics, tocolysis and bedrest, may be more effective than previously thought [12,13], with neonatal survival of up to 96% with cerclage as compared to 57% without it [13]. Further studies are needed to determine if serial cervical length measurements plus emergency cerclage if needed, is as safe a policy as prophylactic cerclage in high risk cases.

## Conclusion

Despite all the controversy with respect to cervical insufficiency and cerclage, for women with a classic or characteristic history a prophylactic cervical cerclage may still be the best option. In contrast, in low risk cases prophylactic cerclage is not useful. Recent studies suggest that emergency cerclage plus antibiotics, tocolysis, and bedrest, has a better chance to increase neonatal survival than previously thought. Further randomized studies are needed to determine the effectiveness of cervical cerclage in women at high risk of fetal loss, but such studies are likely to be hampered by difficulty to obtain informed consent. In addition, there is a need for more basic knowledge of cervical ripening, objective assessment of cervical visco-elastic properties, and randomized controlled trials of technical aspects of cervical cerclage (e.g. suturing technique).

## Competing interests

The author declares that he has no competing interests.
